# Construction of Core–Shell CoMoO_4_@γ-FeOOH Nanosheets for Efficient Oxygen Evolution Reaction

**DOI:** 10.3390/nano12132215

**Published:** 2022-06-28

**Authors:** Huijun Song, Jingjing Li, Guan Sheng, Yinling Zhang, Ahmad Azmin Mohamad, Juan Luo, Zhangnan Zhong, Wei Shao

**Affiliations:** 1 State Key Laboratory Breeding Base of Green Chemistry Synthesis Technology, College of Chemical Engineering, Zhejiang University of Technology, Hangzhou 310014, China; huijunsong@126.com (H.S.); 18855492938@163.com (J.L.); 2111901117@zjut.edu.cn (Y.Z.); luojuang2021@163.com (J.L.); zhongzhangnan@126.com (Z.Z.); 2 School of Materials and Mineral Resources Engineering, Universiti Sains Malaysia, Nibong Tebal 14300, Penang, Malaysia; shengguan@student.usm.my (G.S.); aam@usm.my (A.A.M.)

**Keywords:** γ-FeOOH nanosheets, CoMoO_4_@γ-FeOOH heterojunction, oxygen evolution reaction

## Abstract

The oxygen evolution reaction (OER) occurs at the anode in numerous electrochemical reactions and plays an important role due to the nature of proton-coupled electron transfer. However, the high voltage requirement and low stability of the OER dramatically limits the total energy converting efficiency. Recently, electrocatalysts based on multi-metal oxyhydroxides have been reported as excellent substitutes for commercial noble metal catalysts due to their outstanding OER activities. However, normal synthesis routes lead to either the encapsulation of excessively active sites or aggregation during the electrolysis. To this end, we design a novel core–shell structure integrating CoMoO_4_ as support frameworks covered with two-dimensional γ-FeOOH nanosheets on the surface. By involving CoMoO_4_, the electrochemically active surface area is significantly enhanced. Additionally, Co atoms immerge into the γ-FeOOH nanosheet, tuning its electronic structure and providing additional active sites. More importantly, the catalysts exhibit excellent OER catalytic performance, reducing overpotentials to merely 243.1 mV a versus 10 mA cm^−2^. The current strategy contributes to advancing the frontiers of new types of OER electrocatalysts by applying a proper support as a multi-functional platform.

## 1. Introduction

Developing renewable energy technologies is of great significance for lowering the growing rate of energy consumption and mitigating the deterioration of the living environment [1,2,3,4,5,6]. However, the sustainable energy sources, primarily solar and wind, have so far impeded the large-scale practical application of renewable energy. To solve this issue, electrocatalytic water splitting, consisting of an oxygen evolution reaction (OER) and a hydrogen evolution reaction (HER), is a promising approach to generating sustainable H_2_ from water without using additional reactants. However, the sluggish kinetics of the OER at the anode involves a complex four-step proton-coupled electron transfer process and is regarded as a thermodynamic- and kinetic rate-limiting reaction. Therefore, it is essential to explore efficient electrocatalysts that can effectively facilitate an OER. Even though Ir-/Ru-based oxides show outstanding electrocatalytic behaviors for OER, the high cost and scarcity of these noble catalysts significantly impedes their large-scale applications. To this end, the development of efficient OER catalysts that are inexpensive, easily accessible, and demonstrate excellent electrocatalytic performance (exhibiting a lower overpotential, a high Faradaic efficiency, and adequate catalytic stability) as substitutes for noble metal-based catalysts is both highly important and urgent.

Newly developed OER catalysts can be classified into two types. Metal-free nanomaterials such as carbon nanotube (CNT), graphene, and boron nitride are reported as efficient OER electrocatalysts; however, these catalysts always require multi-step functionalization and are sensitive to pH in the system [7,8]. On the other hand, first-row transition metal- (Mn, Fe, Co, Ni, etc.) based OER catalysts (such as layered double hydroxides (LDHs) [9,10], metal oxides [11], phosphides [12], selenides [13], sulfides [14], and nitrides [15]) have attracted enormous attention as next-generation OER catalysts due to their abundance. These transition metal catalysts have proven to exhibit outstanding OER performances; however, recent works suggest that most of these OER catalysts will undergo structural self-reconstruction during the electrolysis process [16]. More specifically, oxyhydroxides are inevitably formed through the electro-oxidization of these pre-catalysts, which are identified as the real active sites for OER. Nevertheless, most self-reconstructions of pre-catalysts generally occur exclusively on the surface of pre-catalysts, leading to the formation of the typical core–shell (pre-catalysts@oxyhydroxides) structures, in which large amounts of inactive reaction sites are buried in the core [17]. In addition, a sophisticated process and additional amounts of energy consumption are usually required for the re-construction of pre-catalysts. Thus, exploring the direct wet-chemical synthesis of oxyhydroxides with fantastic performance for OERs is of great importance. The layered γ-type iron oxyhydroxide (γ-FeOOH) has been reported to exhibit superior OER performance; however, its layered crystal structure and sheet-shaped morphology tend to make γ-FeOOH nanosheets aggregate because of the high surface energy, which severely diminishes the number of exposed active sites [18]. An effective strategy to overcome this drawback involves constructing heterostructures in which the arrayed γ-FeOOH nanostructures are decorated on other nanomaterials as a substrate with a robust structural stability. Moreover, the desired interfaces between the nanostructures could regulate the electron configuration of the active site as well as the activity owing to the coupling interfaces and the synergistic effect of the heterostructures. For example, the trimetallic phosphate (Fe–Co–Ni–P-1) has been reported to exhibit faster kinetics and a better long-term stability in the catalytic process, which is mainly attributed to monodisperse core–shell structure-induced low resistance, the large electrochemically active area, and the synergistic effect among metals [19]. Wang’s group has recently reported that the core–shell structure of Au@NiCo_2_S_4_ core–shell NPs could result in a higher proportion of high-valance Ni/Co cations and improve its electronic conductivity, which collectively enhances its OER catalytic performance [20]. In addition, a great number of the heterostructures have also been fabricated for an improved electronical performance, such as CoP/FeP_4_ [21], Co_9_S_8_/CoO/NC [22], CoFeS_2_@CoS_2_ [23], Ni-P-S@FeOOH/CC [24], Ni_5_P_4_/Ni_2_P–FeNi [25], and MnO-OVs/NCNTs [26].

Considering the above discussion, we develop herein a facial strategy to construct core–shell CoMoO_4_@γ-FeOOH nanosheets grown on nickel foam by the interface engineering strategy. The obtained CoMoO_4_@γ-FeOOH core–shell nanosheet structures are composed of arrayed γ-FeOOH nanosheets anchored on CoMoO_4_ nanosheets. The synergistic effect provides the CoMoO_4_@γ-FeOOH core–shell nanosheets with a highly electrochemically active surface area due to the core CoMoO_4_‘s support as well as highly and intrinsically active sites with a Co atom-modified γ-FeOOH shell layer. The designed CoMoO_4_@γ-FeOOH nanosheets exhibit a remarkable OER catalytic performance, requiring overpotentials of only 243.1 and 278.5 mV to obtain 10 and 100 mA cm^−2^, respectively, with a negligible degradation after 36 h of OER electrolysis at multi current densities.

## 2. Materials and Methods

### 2.1. Reagents and Chemicals

Cobalt nitrate hexahydrate (Co(NO_3_)_2_·6H_2_O), sodium molybdate dihydrate (Na_2_MoO_4_·2H_2_O), potassium ferrate(K_2_FeO_4_), sodium hydroxide NaOH, and acetone were analysis reagents (AR) and purchased from Aladdin Biochemical Technology Co. Ltd. (Shanghai, China). Ethanol and hydrogen chloride (HCl) were of AR grade and obtained from Shanghai Macklin Biochemical Technology Co., Ltd. (Shanghai, China). Ruthenium oxide (RuO_2_) was provided by Sigma-Aldrich (St. Louis, MO, USA).

### 2.2. Methods

For the synthesis of Ni foam supported CoMoO_4_, 0.175 g Co(NO_3_)_2_·6H_2_O (0.6 mmol) and 0.145 g Na_2_MoO_4_·2H_2_O (0.6 mmol) were dissolved in 5 mL deionized water (DI water) under sonication. These two transparent solutions were mixed under continuous stirring for 0.5 h and then transferred into a 25 mL Teflon-lined autoclave. A piece of Ni foam (1.0 × 3.0 cm^2^) was thoroughly rinsed with 1.0 M HCl, acetone, and ethanol in sequence under sonication in a 20 mL glass vial. The pretreated Ni foam was put into the autoclave mentioned above, and then the mixed system was kept at 150 °C for 4 h. The Ni foam samples were taken out, washed, and vacuum dried, and CoMoO_4_ arrays were obtained.

For the synthesis of Ni foam supported CoMoO_4_@γ-FeOOH, 7.5 mM K_2_FeO_4_ solution containing 6 M NaOH was firstly prepared by dissolving K_2_FeO_4_ into aqueous NaOH solution under sonication and then centrifuged at 9000 rpm for 1 min. Subsequently, the as-prepared CoMoO_4_ was soaked into 20 mL of the above solution. After reacting for 24 h at ambient conditions without any disruption, the obtained CoMoO_4_@γ-FeOOH was cleaned with deionized water and then dried naturally. The Ni foam supported γ-FeOOH was also synthesized via a similar procedure, except for the fact that the CoMoO_4_ was replaced with pretreated Ni foam.

### 2.3. Materials Characterizations

Scanning electron microscope (SEM) images were collected with a field-emission scanning electron microscope ZEISS-G500 (ZEISS, Jena, Germany) at an accelerating voltage of 5 kV. The as-synthesized samples were scraped from the Ni foam and analyzed with a Titan Themis Z (Thermo Fisher Scientific, Waltham, MA, USA) to record high-resolution TEM (HRTEM) images, elemental mapping, and SAED patterns. The Raman spectra of all synthesized samples were obtained using a HORIBA Jobin–Yvon Lab-Ram ARAMIS (Horiba, Kyoto, Japan) Raman spectrometer equipped with a CCD detector. X-ray diffraction, D8Advance (Bruker, Billerica, MA, USA) was used for the crystalline phase analysis with Cu Kα radiation. The surface properties of all samples were characterized using X-ray photoelectron spectroscopy (XPS) ESCALAB 250, (Thermo Fisher Scientific, Waltham, MA, USA). All binding energies were calibrated with the C 1 s peak at 284.8 eV corresponding to adventitious carbon found on the surface.

### 2.4. Electrochemical Measurements

All the electrochemical tests were conducted in fresh 1.0 M KOH on a CHI 760E electrochemical station using a standard three-electrode system. The as-prepared catalysts that were grown on the Ni foam (0.5 × 1.0 cm^2^) directly served as the working electrode. The standard Hg/HgO electrode was applied as the reference electrode, while a platinum wire was used as the counter electrode. All the measured potentials were calibrated to a reversible hydrogen electrode (RHE): RHEs: E_RHE_ = E_Hg/HgO_ + 0.059 × pH + 0.098 (V). Linear sweep voltammetry (LSV) with 95% iR compensation was carried out at a scan rate of 0.5 mV s^−^^1^. Multicurrent densities were applied for measuring long-term electrochemical stability. The double-layer capacitance (C_dl_) was estimated based on the cyclic voltammograms (CVs) recorded at various scan rates over a non-faradaic potential range of 1.024–1.124 V (E_RHE_).

## 3. Results and Discussion

**Figure 1:**
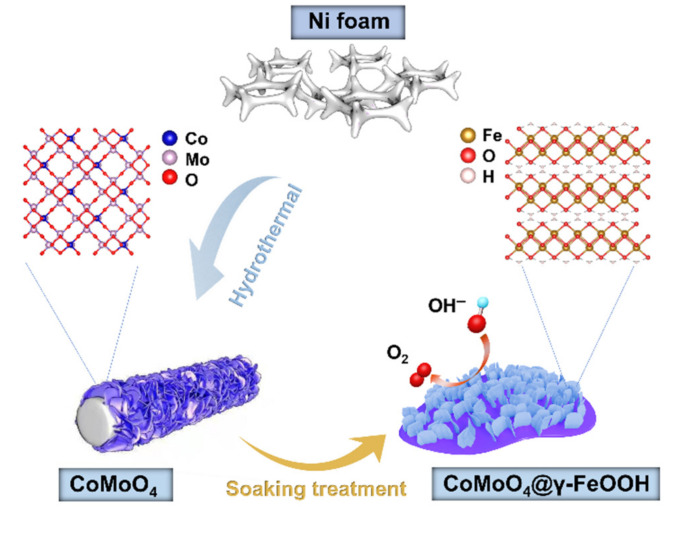
Schematic illustration for the construction of CoMoO_4_@γ-FeOOH OER catalyst.

**Figure 2:**
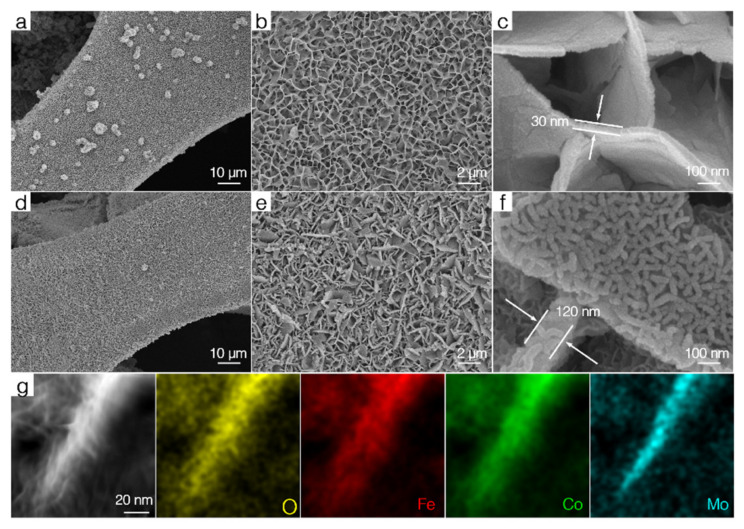
SEM images of (**a**–**c**) CoMoO_4_ and (**d**–**f**) CoMoO_4_@γ-FeOOH. (**g**) HAADF-STEM and the corresponding EDS mapping images of CoMoO_4_@γ-FeOOH.

Figure 1 illustrates the procedure for constructing Ni foam-supported hierarchical 2D core–shell CoMoO_4_@γ-FeOOH nanosheets. In brief, the CoMoO_4_ nanosheets were uniformly grown on Ni foam through a reported hydrothermal method with some modifications, followed by an immersion treatment with a mixed solution containing K_2_FeO_4_ and NaOH at room temperature for 24 h to prepare the core–shell structured CoMoO_4_@γ-FeOOH nanosheets. This core–shell structure could effectively prevent the high surface energy of γ-FeOOH nanosheet-induced aggregation, which could increase the number of exposed active sites and the electrochemically active surface area (ECSA) of γ-FeOOH nanosheets. Scanning electron microscopy (SEM) images showed the morphological and structural evolution process of the as-prepared catalysts. As shown in Figure 2a–c, the as-prepared CoMoO_4_ presents micrometer-length nanosheets similar to the reported results [27]. In addition, the as-prepared CoMoO_4_ nanosheets on the Ni foam surface displayed a uniform coverage, and the thickness of a single nanosheet was about ~30 nm. After reacting in the solution containing K_2_FeO_4_ and NaOH, the SEM images (Figure 2d–f) show that the surface of the CoMoO_4_ nanosheets turned rough and was decorated with some worm-like γ-FeOOH nanomaterials. In addition, the thickness of the CoMoO_4_ increased to 120 nm. A high-angle annular dark-field scanning transmission electron microscopy (HAADF-STEM) image (Figure 2g) shows that these worm-like γ-FeOOH nanomaterials are actually tiny nanosheets. The corresponding energy-dispersive X-ray spectroscopy (EDS) mapping images of a vertical CoMoO_4_@γ-FeOOH nanosheet clearly show that the Mo and Fe elements are mainly distributed in the core and shell of the CoMoO_4_@γ-FeOOH nanosheets, respectively, while the Co and O elements are homogeneously distributed over the entire hierarchical 2D core–shell CoMoO_4_@γ-FeOOH nanosheets. To further elucidate the element distribution of the shell (tiny γ-FeOOH nanosheets) of the CoMoO_4_@γ-FeOOH hierarchical nanosheets, the EDS mapping of horizonal CoMoO_4_@γ-FeOOH hierarchical nanosheets and a line scan were performed. As shown in Figure S1, Fe has the largest dispersion area and exhibits more content than Co on the edge of the tiny γ-FeOOH, and there is almost no Mo signal on the tiny γ-FeOOH nanosheet. However, the content of Co is higher than Fe in the interior of the tiny γ-FeOOH, suggesting the probable ion exchange of Co and Fe during the formation of γ-FeOOH. Such ion exchange reactions could promote the nucleation of γ-FeOOH on the surface of CoMoO_4_ and benefit the formation of a core–shell structure of CoMoO_4_@γ-FeOOH. The pure γ-FeOOH nanosheets supported on the Ni foam were also prepared as the monophasic counterpart. The SEM images (Figure S2a–c) show that the tiny γ-FeOOH nanosheets were vertically and uniformly grown on the Ni foam, exhibiting a similar morphology to the shell of the CoMoO_4_@γ-FeOOH hierarchical nanosheets. Nevertheless, the pure γ-FeOOH powder synthesized without the Ni foam substrate exhibited a highly stacked morphology (Figure S2d).

**Figure 3:**
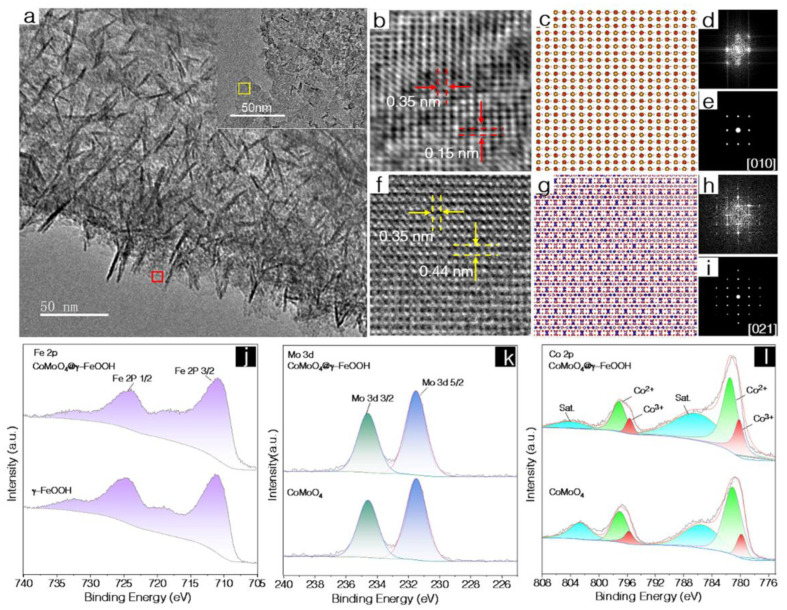
(**a**) Low magnification TEM image of CoMoO_4_@γ-FeOOH’s heterostructure, insets show the γ-FeOOH region (red rectangle), and CoMoO_4_ region (yellow rectangle). (**b**) Magnified HRTEM view of the crystalline region of γ-FeOOH. (**c**) The crystal structure model of γ-FeOOH observed along the [010] axis. (**d**) FFT of the (**b**,**e**) simulated FFT of the crystal structure model of γ-FeOOH observed along the [010] axis. (**f**) Magnified HRTEM view of the crystalline region of CoMoO4. (**g**) The crystal structure model of CoMoO4 observed along the [021] axis. (**h**) FFT of the (**f**,**i**) simulated FFT of the crystal structure. (**j**) High-resolution Fe 2p XPS spectra of CoMoO_4_@γ-FeOOH and CoMoO_4_. (**k**) High-resolution Mo 3d XPS spectra of CoMoO_4_@γ-FeOOH and CoMoO_4_. (**l**) High-resolution Co 2p XPS spectra of CoMoO_4_@γ-FeOOH and γ-FeOOH.

A powder X-ray diffraction (PXRD) was carried out to investigate the structural information. As shown in Figure S3a, the main diffraction peaks of the CoMoO_4_ nanosheets could be assigned to CoMoO_4_ (PDF no. 25-1434). Following the reaction in the mixed solution containing K_2_FeO_4_ and NaOH, no apparent new diffraction peaks were observed in the CoMoO_4_@Fe based nanosheets compared with that of CoMoO_4_, suggesting that γ-FeOOH possessed a lower crystallinity and that the core retained the structure of CoMoO_4_. The pure γ-FeOOH was also characterized (Figure S3b) and several weak diffraction peaks that adequately matched the γ-FeOOH could be found [28]. To furtherly confirm the formation of CoMoO_4_@γ–FeOOH, Raman tests were conducted. As shown in Figure S4, the bands located at 326, 846, and 935 cm^−1^ for the Co–O–Mo, O–Mo–O, and Mo–O vibrations, respectively, could be found in both the CoMoO_4_ and CoMoO_4_@γ-FeOOH nanosheets [29], while the Raman band located at 230, which could likely be ascribed to Fe–O vibrations, could be found in both the γ-FeOOH and CoMoO_4_@γ-FeOOH nanosheets [18]. This spectra information indicates the co-existence of CoMoO_4_ and γ-FeOOH in the CoMoO_4_@γ-FeOOH nanosheets. The HRTEM imaging allowed for the atomic-scale structural identification of CoMoO_4_@γ-FeOOH nanosheets. Figure 3a shows the low magnification TEM image of the CoMoO_4_@γ-FeOOH nanosheets, from which we can tell that its morphology comprises a bunch of intersecting layers. The yellow rectangular area in the inset is the CoMoO_4_ component and the red one represents the γ-FeOOH nanosheets growing upon the much larger CoMoO_4_ sheets as the matrix. The HRTEM image (Figure 3b) and its corresponding diffractogram (Figure 3d) after the implementation of the Fast Fourier Transform (FFT) of the γ-FeOOH nanosheets reveal the {200} planes where the lattice spacing is 1.5 Å and the {002} planes where the lattice spacing is 3.5 Å; the nanosheets’ identification was confirmed from crystallography since it adequately matches the crystalline model of γ-FeOOH observed along the [010] axis (Figure 3c) and the corresponding simulated FFT pattern (Figure 3e). Similar to what was mentioned previously, the HRTEM image and corresponding Fast Fourier transform pattern (FFT) in Figure 3f,h indicate the {$11\bar{2}$} planes where the lattice spacing is 3.5 Å and the {200} planes where the lattice spacing is 4.4 Å for CoMoO_4_ observed along the [021] axis, as can be indexed from the combination of Figure 3g and its simulated corresponding FFT (Figure 3i).

Previous reports have demonstrated that the electronic structure of an active site is highly related to the catalytic activity and could be adequately modulated by constructing heterostructures [30,31]. Hence, X-ray photoelectron spectroscopy (XPS) was employed to study detailed atomic and electronic structural information. As shown in Figure S5a, the survey spectrum of CoMoO_4_@γ-FeOOH shows the coexistence of Co, Fe, Mo, O, and C, indicating the successful growth of γ-FeOOH on the CoMoO_4_ nanosheets. In the high-resolution XPS spectra of Fe 2p (γ-FeOOH and CoMoO_4_@γ-FeOOH), the two peaks located at about 711.2 and 724.7 eV can be attributed to the Fe^2+^ 2p 3/2 and Fe^2+^ 2p 1/2 of Fe-O species [32], respectively (Figure 3j). In addition, similar XPS fine spectra of Mo in the CoMoO_4_ and CoMoO_4_@γ-FeOOH collectively suggests the similar coordination environments of Fe ions and Mo ions in the CoMoO_4_@γ-FeOOH and the corresponding single components (Figure 3k). The peaks at about 780.7 eV (2p 3/2) and 796.8 eV (2p 1/2) in the spectra of Co are consistent with the Co–O species [33] (Figure 3l). However, the fitted results indicate that the peak ratio of Co^2+^/Co^3+^ decreases after decorating γ-FeOOH on the surface of the CoMoO_4_ nanosheets, indicating that the ion exchange of the Co and Fe ions in the γ-FeOOH resulted in the formation of Co-doped γ-FeOOH with partial Co atoms possessing a higher valence. It has been widely reported that active sites with a higher valence could enhance the chemisorption of OH^−^, complement the formation of MOOH (M represents metal, such as Co or Ni) through nucleophilic attack during OER, and are generally regarded as the active sites for OER [34]. Therefore, the as-synthesized biphasic CoMoO_4_@γ-FeOOH nanosheets offer a great opportunity to evoke synergistic electrocatalysis towards OER.

**Figure 4:**
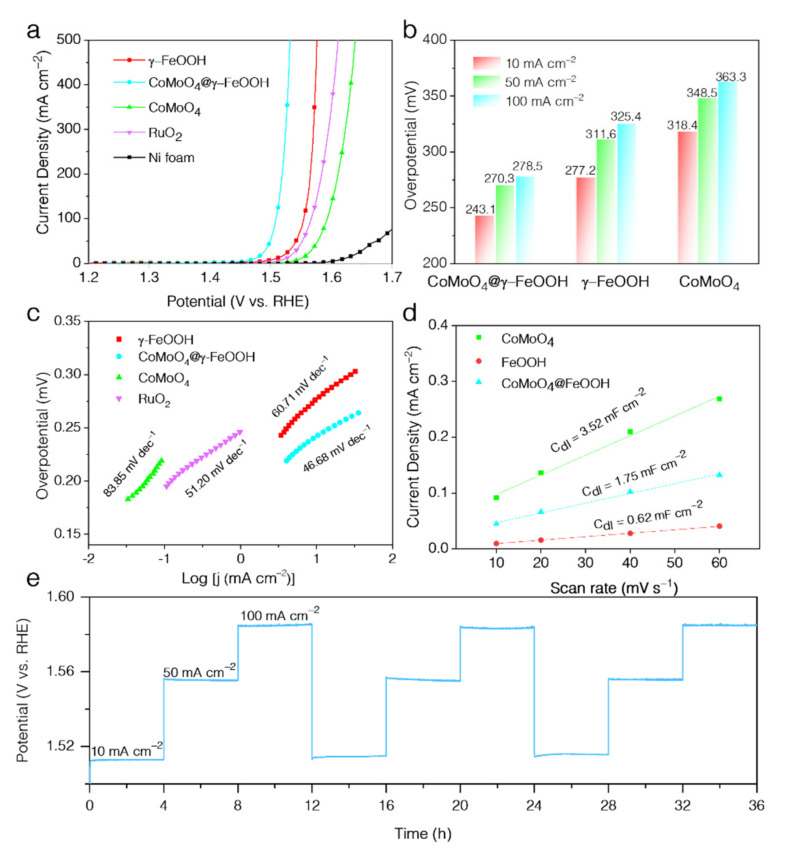
(**a**) LSV curves of CoMoO_4_, CoMoO_4_@γ-FeOOH, γ-FeOOH, RuO_2_, and Ni foam in 1.0 M KOH. (**b**) Comparison of the overpotentials at 10 mA cm^−2^, 50 mA cm^−2^, and 100 mA cm^−2^ for CoMoO_4_, CoMoO_4_@γ-FeOOH, and γ-FeOOH. (**c**) Tafel plots of CoMoO_4_, CoMoO_4_@γ-FeOOH, γ-FeOOH, and RuO_2_. (**d**) CV current density versus scan rate of CoMoO_4_, CoMoO_4_@γ-FeOOH, and γ-FeOOH. (**e**) Durability test of CoMoO_4_@γ-FeOOH in 1.0 M KOH at constant 10 mA cm^−2^, 50 mA cm^−2^, and 100 mA cm^−2^.

The electrochemical behaviors of the as-prepared and commercial catalysts were evaluated with a three-electrode system in O_2_-saturated 1 M KOH electrolytes. To evaluate the OER activity of these materials, RuO_2_ was deposited on the Ni foam as a reference with the same loading. The linear sweep voltammetry (LSV) curves (Figure 4a) with iR-compensation show that the CoMoO_4_@γ-FeOOH catalyst exhibits the most optimal performance, with an outstanding OER current density of 10 mA cm^−2^ with a low overpotential of 243.1 mV, which is 34.1, 75.3, 48.9, and 146.9 mV lower than that of γ-FeOOH, CoMoO_4_, RuO_2_, and Ni foam (Figure 4a,b). Furthermore, very small overpotentials of 270.3 and 278.5 mV are enough to reach a high current density of 50 mA cm^−2^ and 100 mA cm^−2^ for the CoMoO_4_@γ-FeOOH electrode, which is highly competitive when compared to other previously reported OER electrocatalysts (Supplementary Table S1) [35,36,37,38,39,40,41,42,43,44,45,46,47]. Figure 4c shows the Tafel plots of the sample catalysts, where the CoMoO_4_@γ-FeOOH presented the lowest Tafel slope of 46.58 mV dec^−1^ in contrast to those of γ-FeOOH (60.71 mV dec^−^^1^), RuO_2_ (51.20 mV dec^−1^), and CoMoO_4_ (83.85 mV dec^−1^), indicating the rapid kinetics of CoMoO4@γ-FeOOH. The electrochemically active surface area (ECSA) was evaluated by the electrochemical double-layer capacitance (C_dl_) (Figure S6). As seen in Figure 4d, the CoMoO_4_ exhibits a larger double-layer capacitance (C_dl_) value (3.52 mF cm^−2^) than those of CoMoO_4_@γ-FeOOH (1.75 mF cm^−^^2^) and γ-FeOOH (0.62 mF cm^−2^), indicating that CoMoO_4_ could complement the exposure of the active site and largely enhance the ECSA of CoMoO_4_@γ-FeOOH. Comparing the results, the γ-FeOOH exhibits a much higher activity relative to CoMoO_4_ towards OER, suggesting that the Co and Fe sites in the Co-doped γ-FeOOH are both performing as real OER active sites, and that the presence of CoMoO_4_ modifies the environmental conditions of the active sites. Therefore, the core–shell structure of CoMoO_4_@γ-FeOOH not only improves the ECSA of active components by moderating the dispersion of γ-FeOOH, but also enhances the intrinsic activity of γ-FeOOH through the doping of Co atoms, which collectively leads to the higher electronic activity and lower overpotential of CoMoO_4_@γ-FeOOH compared to the single components. The stability was also evaluated via chronopotentiometry experiments without iR compensation for three constant cycles that comprised a test duration of 36 h at multicurrent densities (Figure 4e). The overpotential remained stable for all the cycles; about 283.1, 325.7, and 355.0 mV were required to reach the current densities of 10, 50, and 100 mA cm^−2^, respectively, indicating that the CoMoO_4_@γ-FeOOH heterostructure possesses a superb durability. Faradaic efficiency measurements were also tested to assess the difference between the actual efficiency and the theoretical efficiency of the CoMoO_4_@γ-FeOOH electrode (Figure S7). The detailed test and the calculated method could be referenced with the reported literature [48]. The results exhibited a Faradaic efficiency of about 93.9% after applying a current density of 10 mA cm^−2^ for 5 h, suggesting that all the applied electrons were almost completely used for water oxidation under the consideration of an experimental error.

**Figure 5:**
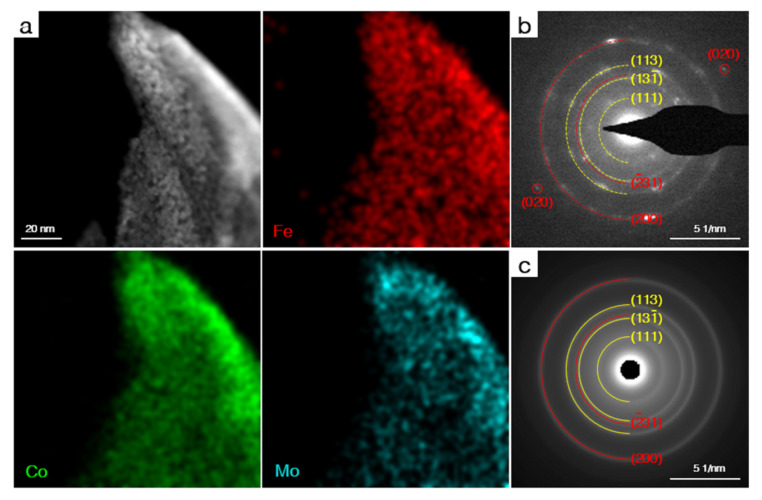
(**a**) HAADF STEM and corresponding EDS mapping images of CoMoO_4_@γ-FeOOH after LSV test. (**b**) SAED and (**c**) Radially-averaged patterns of CoMoO_4_@γ-FeOOH after LSV test.

It has been widely demonstrated that most of the transition metal-based electrocatalysts will undergo a structural transformation during the OER process. Therefore, detailed structure tests were carried out for the catalysts after the LSV test to explore the intrinsic origins of the high OER activity. As shown in Figures S8 and S9, the morphology of CoMoO_4_ and γ-FeOOH showed limited changes after the LSV test, while the morphology of CoMoO_4_@γ-FeOOH exhibited obvious changes, and the γ-FeOOH nanosheets on the CoMoO_4_ turned into some nanoparticles that closely embedded into the surface of CoMoO_4_. The Raman and selected area electron diffraction (SAED) methods were used to investigate the structural evolution of CoMoO_4_@γ-FeOOH after the LSV test. As shown in Figure S10, the CoMoO_4_@γ-FeOOH after the LSV test showed typical peaks for Fe-O, Co-O-Mo, and Mo-O vibrations that were similar to the initial Raman spectrum of CoMoO_4_@γ-FeOOH. The HAADF STEM mapping (Figure 5a) results revealed that the CoMoO_4_@γ-FeOOH after the LSV test retained its initial element composition and exhibited a homogeneous dispersion of the Mo, Co, and Fe atoms. Detailed phase information was collected through SAED. The SAED pattern (Figure 5b) exhibits a typical diffraction pattern characteristic of polycrystalline materials, of which the diffraction pattern and the radially-averaged diffraction rings (Figure 5c) can be adequately indexed to both the CoMoO_4_ (marked with yellow wires) and γ-FeOOH phases (marked with red wires) of the heterostructure. The XPS spectra of CoMoO_4_@γ-FeOOH after the LSV test showed a similar elemental composition and valence (Figure S11). These limited changes in the chemical environment and phase composition indicate that the CoMoO_4_@γ-FeOOH adequately preserved its initial structure under OER conditions, which may largely contribute to its superb stability in OER reactions.

## 4. Conclusions

In conclusion, we have demonstrated a general strategy for constructing highly efficient OER catalysts through the post-synthesis of muti-metal oxyhydroxides on stable support frameworks. An HRTEM was employed to successfully confirm the synthesis of a sheet-like CoMoO_4_@γ-FeOOH core–shell structure, and EDS mapping proved that the Co atoms immersed into the γ-FeOOH to form dual-metal entities. The as-prepared catalysts show a distinguished OER performance with a low overpotential of 243.1 mV under a current density of 10 mA cm^−2^. Moreover, the CoMoO_4_@γ-FeOOH presented a very low Tafel slope of 46.58 mV dec^−1^, as well as a very highly electrochemically active surface area. We further investigated the catalyst structure and composition after 36 h of electrolysis, which demonstrated a very high stability in accordance with our design purpose.

## Data Availability

The data that support the findings of this study are available from the corresponding authors upon reasonable request.

## Supplementary Materials

The following supporting information can be downloaded at: https://www.mdpi.com/article/10.3390/nano12132215/s1, Figure S1: (a) HAADF STEM and EDS mapping images of CoMoO_4_@γ-FeOOH. (b) EDS Line scan spectrum of the area marked with blue arrow in (a). Figure S2: (a) Low-and (b,c) high-magnification SEM images of γ-FeOOH, (d) TEM image of γ-FeOOH powder synthesized without Ni foam substrate. Figure S3: (a) XRD patterns of CoMoO_4_ and CoMoO_4_@γ-FeOOH, (b) XRD pattern of γ-FeOOH. Figure S4: Raman spectra of CoMoO_4_, CoMoO_4_ @γ-FeOOH, and γ-FeOOH. Figure S5: XPS survey spectra of (a) CoMoO_4_@γ-FeOOH, (b) CoMoO_4_, and (c) γ-FeOOH. Figure S6: CV curves of (a) CoMoO_4_, (b) CoMoO_4_@γ-FeOOH, and (c) γ-FeOOH acquired at various scan rates. Figure S7: Faradaic efficiency of CoMoO_4_@γ-FeOOH for the theoretically calculated and experimentally measured O_2_ at a current density of 10 mA cm^−2^. Figure S8: SEM images of catalysts after LSV test, (a–c) CoMoO_4_, (d–f) γ-FeOOH. Figure S9. (a–c) SEM images of CoMoO_4_@γ-FeOOH after LSV test with different magnifications. Figure S10: Raman spectra of CoMoO_4_@γ-FeOOH after LSV test. Figure S11: (a) XPS survey spectrum, (b) high-resolution Co 2p XPS spectrum, (c) high-resolution Mo 3d XPS spectrum, (d) high-resolution Fe 2p XPS spectrum of CoMoO_4_@γ-FeOOH after LSV test. Table S1: Comparison of OER performances between CoMoO_4_@γ-FeOOH electrode and recently reported electrocatalysts in alkaline solution. References [35,36,37,38,39,40,41,42,43,44,45,46,47] have been cited in the Supplementary Materials.
