# Supplementary material for: Construction of Core–Shell CoMoO4@γ-FeOOH Nanosheets for Efficient Oxygen Evolution Reaction
Source: Nanomaterials (Basel). 2022 Jun 28;12(13):2215. doi: 10.3390/nano12132215 (PMC9268127; doi:10.3390/nano12132215)
Supplement: Supplementary file 1 [file nanomaterials-12-02215-s001.zip › nanomaterials-1777877 - Supplementary Materials.pdf]

nanomaterials

Supplementary Materials

Construction of Core–Shell $CoMoO_4$@γ-FeOOH Nanosheets for Efficient Oxygen Evolution Reaction

Huijun Song [1,†], Jingjing Li [1,†], Guan Sheng [2,†], Yinling Zhang [1], Ahmad Azmin Mohamad [2], Juan Luo [1], Zhangnan Zhong [1] and Wei Shao [1,*]

1 State Key Laboratory Breeding Base of Green Chemistry Synthesis Technology, College of Chemical Engineering, Zhejiang University of Technology, Hangzhou 310014, China; huijunsong@126.com (H.S.); 18855492938@163.com (J.L.); 2111901117@zjut.edu.cn (Y.Z.); luojuang2021@163.com (J.L.); zhongzhangnan@126.com (Z.Z.)
2 School of Materials and Mineral Resources Engineering, Universiti Sains Malaysia, Nibong Tebal 14300, Penang, Malaysia; shengguan@student.usm.my (G.S.); aam@usm.my (A.A.M.)
* Correspondence: weishao@zjut.edu.cn
† These authors contributed equally to this work.

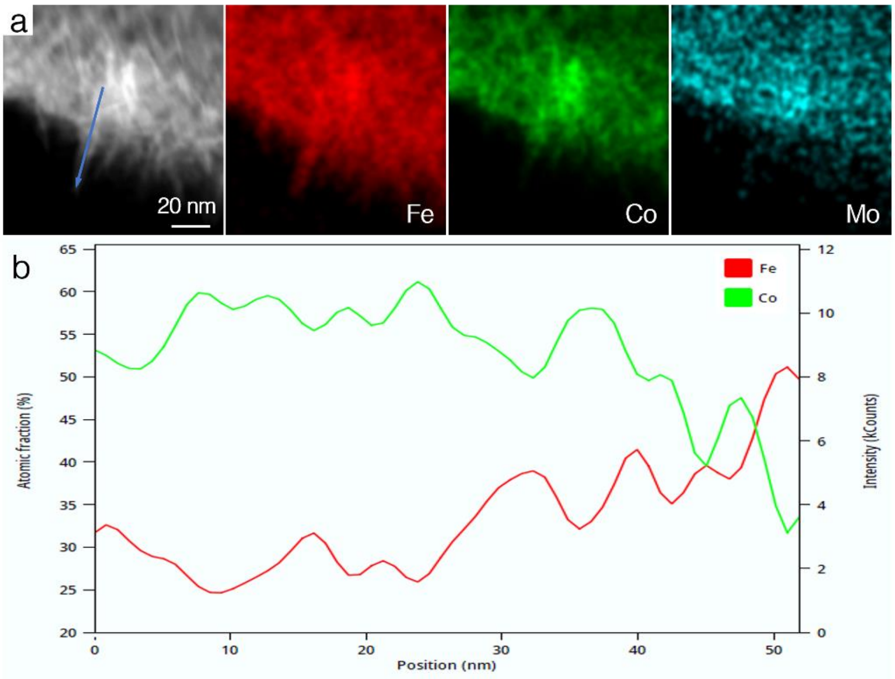

Figure S1. (**a**) HAADF STEM and EDS mapping images of $CoMoO_4$@γ-FeOOH. (**b**) EDS Line scan spectrum of the area marked with blue arrow in (**a**).

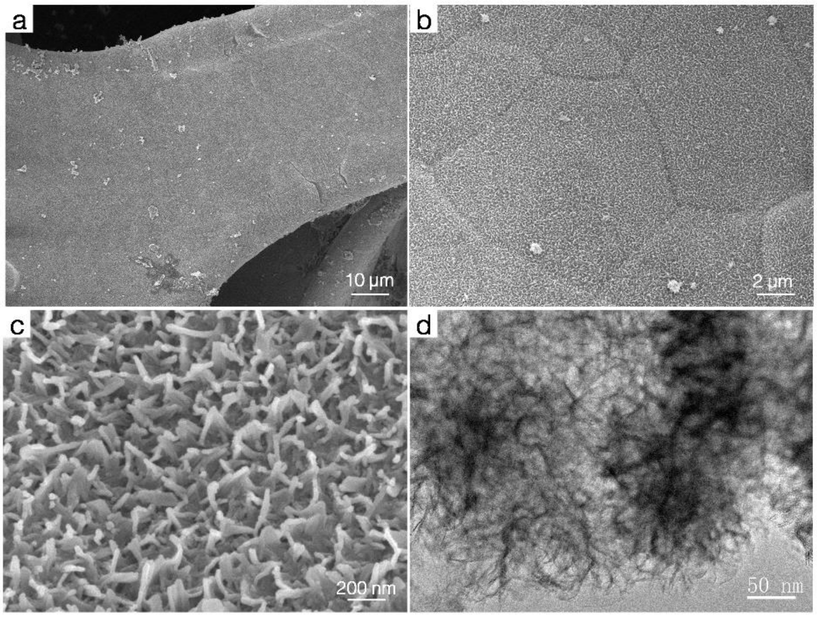

Figure S2. (**a**) Low-and (**b**,**c**) high-magnification SEM images of γ-FeOOH. (**d**) TEM image of γ-FeOOH powder synthesized without Ni foam substrate.

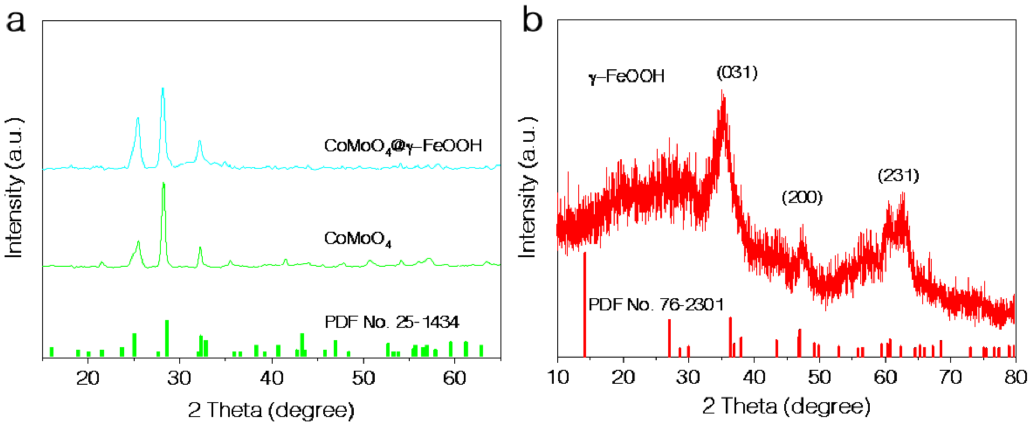

Figure S3. (**a**) XRD patterns of $CoMoO_4$ and $CoMoO_4$@γ-FeOOH. (**b**) XRD pattern of γ-FeOOH.

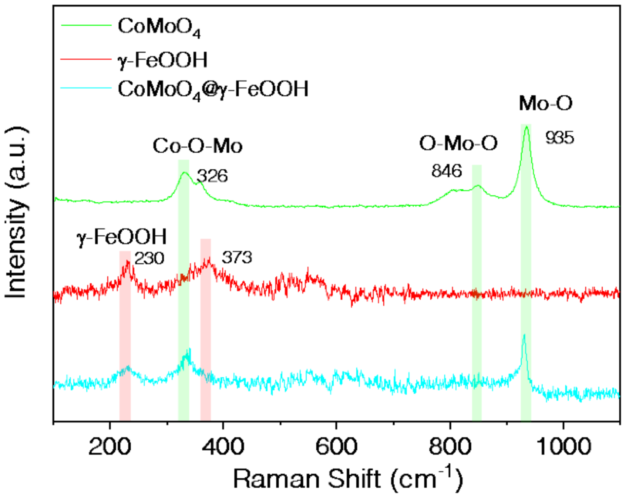

Figure S4. Raman spectra of $CoMoO_4$, $CoMoO_4$@γ-FeOOH and γ-FeOOH.

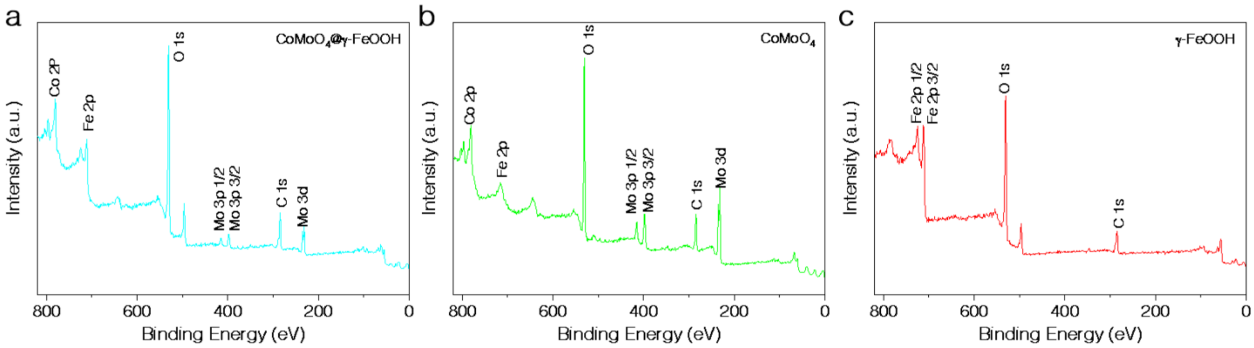

Figure S5. XPS survey spectra of (**a**) $CoMoO_4$@γ-FeOOH, (**b**) $CoMoO_4$ and (**c**) γ-FeOOH.

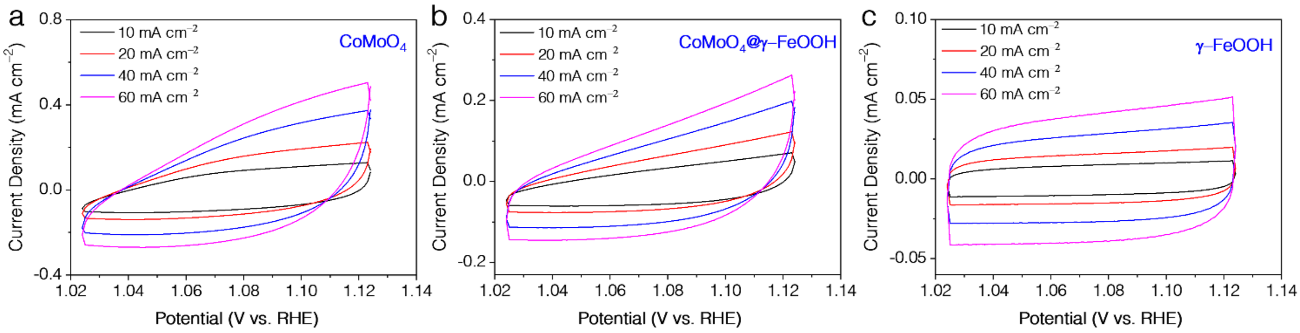

Figure S6. CV curves of (**a**) $CoMoO_4$, (**b**) $CoMoO_4$@γ-FeOOH, and (**c**) γ-FeOOH acquired at various scan rates.

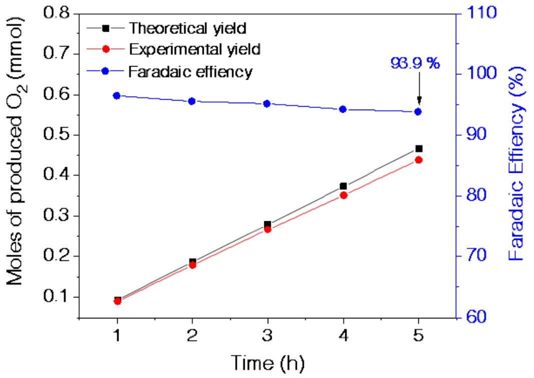

Figure S7. Faradaic efficiency of $CoMoO_4$@γ-FeOOH for the theoretically calculated and experimentally measured O_2 at a current density of 10 mA cm^{-2}.

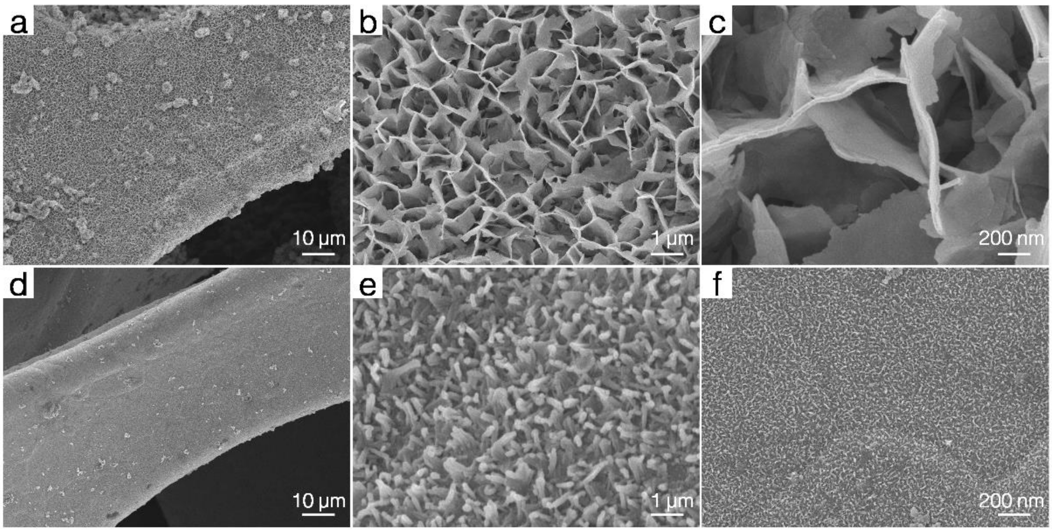

Figure S8. SEM images of catalysts after LSV test; (**a–c**) $CoMoO_4$, (**d–f**) γ-FeOOH.

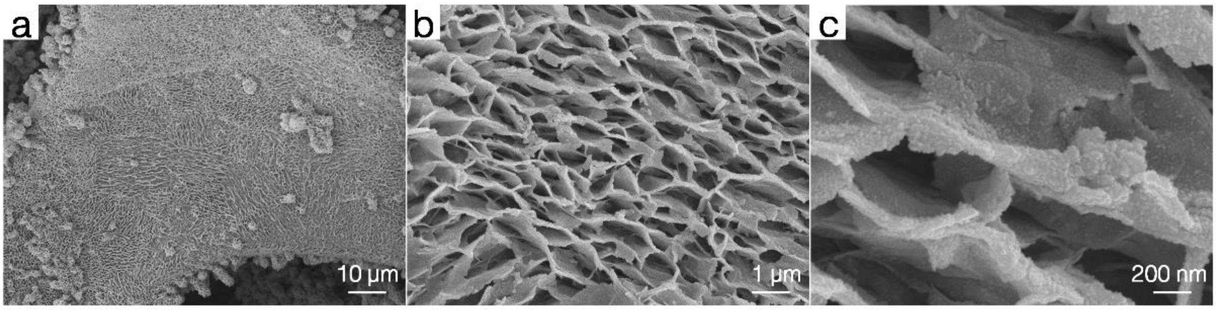

Figure S9. (**a–c**) SEM images of $CoMoO_4$@γ-FeOOH after LSV test.

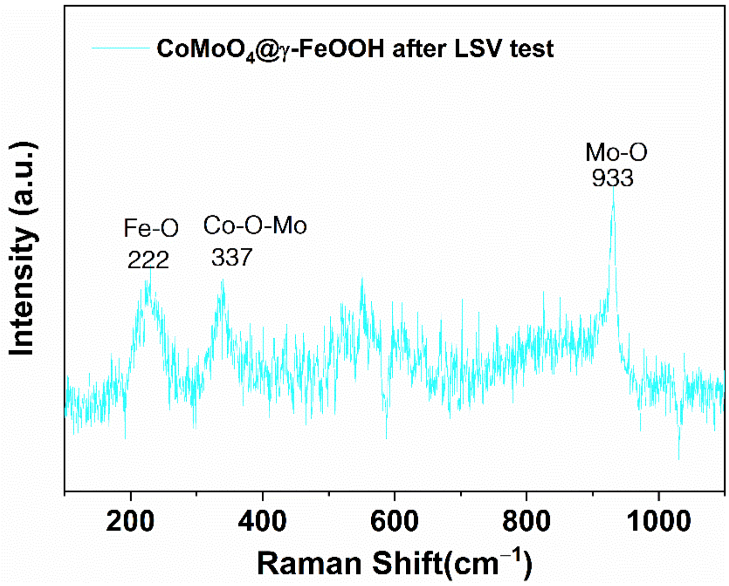

Figure S10. Raman spectra of $CoMoO_4$@γ-FeOOH after LSV test.

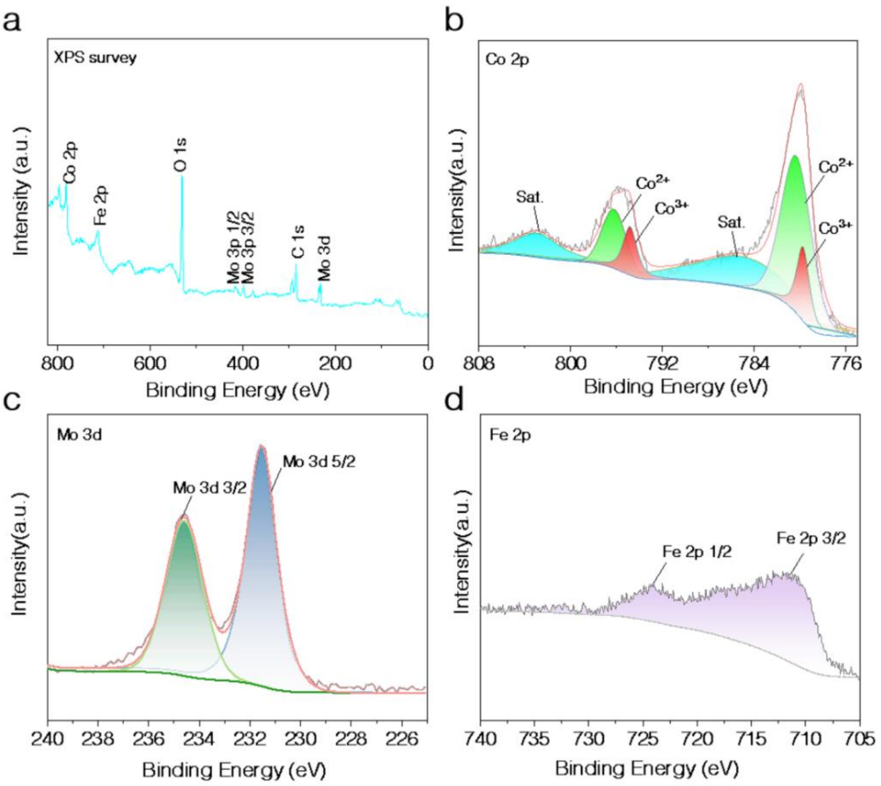

Figure S11. (**a**) XPS survey spectrum, (**b**) high-resolution Co 2p XPS spectrum, (**c**) high-resolution Mo 3d XPS spectrum, (**d**) high-resolution Fe 2p XPS spectrum of $CoMoO_4$@γ-FeOOH after LSV test.

Table S1. Comparison of OER performances between $CoMoO_4$@γ-FeOOH electrode and recently reported electrocatalysts in alkaline solution.

**Catalyst**	**Substrate**	**Overpotential (mV)**	**Tafel Slope (mV dec^{-1})**	**Stability**	**Ref.**
$CoMoO_4$@γ-FeOOH	[a] NF	243, 270, 279 @10, 50, 100 mA cm^{-2}	46.7	36 h@10, 50, 100 mA cm^{-2}	**This work**
$NiFe_2O_{4-x}$/NMO-25	NF	262, 304@10, 100 mA cm^{-2}	42.7	40 h@200 mA cm^{-2}	[1]
Ni_3S_2@MoS_2/FeOOH	NF	260@10 mA cm^{-2}	49	24 h@10mA cm^{-2}	[2]
CoFe LDH-F	NF	300@10 mA cm^{-2}	47	35 h@10 mA cm^{-2}	[3]
Fe-NiO/NF	NF	264, 336@10, 100 mA cm^{-2}	65.3	12 h@60mA cm^{-2}	[4]
Ni_3S_2/MnO_2	NF	260, 348@10, 100 mA cm^{-2}	61	48 h@100 mA cm^{-2}	[5]
Amorphous (Fe-Ni) Co_x-OH/Ni_3S_2	NF	280@100 mA cm^{-2}	57	100 h@200 mA cm^{-2}	[6]
PA-NiO	NF	310@100 mA cm^{-2}	36	7 h@100 mA cm^{-2}	[7]
Mo-NiOOH	NF	390@ 100 mA cm^{-2}	68	24 h@100mA cm^{-2}	[8]
Ni_5Co_3Mo-OH	NF	304@100 mA cm^{-2}	56.4	100 h@100 mA cm^{-2}	[9]
Mo-CoOOH	[b] CC	305, 365@10, 100 mA cm^{-2}	56	20 h@40 mA cm^{-2}	[10]
NiO@Ni/WS_2	CC	380@50 mA cm^{-2}	108.9	40 h@50mA cm^{-2}	[11]
$Mo_{51}Ni_{40}Fe_9$	[c] GCE	257@10 mA cm^{-2}	51	18 h@10 mA cm^{-2}	[12]
CoMoOS NBs	GCE	281@10 mA cm^{-2}	75.4	40 h@10 mA cm^{-2}	[13]

[a] NF; nickel foam, [b] CC; carbon cloth, [c] GCE; glassy carbon electrode.

References

1. Choi, J.; Kim, D.; Zheng, W.; Yan, B.; Li, Y.; Lee, L.Y.S.; Piao, Y. Interface engineered $NiFe_2O_{4-x}/NiMoO_4$ nanowire arrays for electrochemical oxygen evolution. *Appl. Catal. B: Environ.* **2021**, *286*, 119857, https://doi.org/10.1016/j.apcatb.2020.119857.
2. Zheng, M.; Guo, K.; Jiang, W.-J.; Tang, T.; Wang, X.; Zhou, P.; Du, J.; Zhao, Y.; Xu, C.; Hu, J.-S. When MoS_2 meets FeOOH: A "one-stone-two-birds" heterostructure as a bifunctional electrocatalyst for efficient alkaline water splitting. *Appl. Catal. B: Environ.* **2018**, *244*, 1004–1012, https://doi.org/10.1016/j.apcatb.2018.12.019.
3. Liu, P.F.; Yang, S.; Zhang, B.; Yang, H. Defect-Rich Ultrathin Cobalt–Iron Layered Double Hydroxide for Electrochemical Overall Water Splitting. *ACS Appl. Mater. Interfaces* **2016**, *8*, 34474–34481, https://doi.org/10.1021/acsami.6b12803.
4. Qiu, Z.; Ma, Y.; Edvinsson, T. In operando Raman investigation of Fe doping influence on catalytic NiO intermediates for enhanced overall water splitting. *Nano Energy* **2019**, *66*, 104118, https://doi.org/10.1016/j.nanoen.2019.104118.
5. Xiong, Y.; Xu, L.; Jin, C.; Sun, Q. Interface-engineered atomically thin Ni_3S_2/MnO_2 heterogeneous nanoarrays for efficient overall water splitting in alkaline media. *Appl. Catal. B: Environ.* **2019**, *254*, 329–338, https://doi.org/10.1016/j.apcatb.2019.05.017.
6. Che, Q.; Li, Q.; Chen, X.; Tan, Y.; Xu, X. Assembling amorphous (Fe-Ni)Co_x-OH/Ni_3S_2 nanohybrids with S-vacancy and interfacial effects as an ultra-highly efficient electrocatalyst: Inner investigation of mechanism for alkaline water-to-hydrogen/oxygen conversion. *Appl. Catal. B: Environ.* **2019**, *263*, 118338, https://doi.org/10.1016/j.apcatb.2019.118338.
7. Li, Z.; Niu, W.; Zhou, L.; Yang, Y. Phosphorus and Aluminum Codoped Porous NiO Nanosheets as Highly Efficient Electrocatalysts for Overall Water Splitting. *ACS Energy Lett.* **2018**, *3*, 892–898, https://doi.org/10.1021/acsenergylett.8b00174.
8. Jin, Y.; Huang, S.; Yue, X.; Shu, C.; Shen, P.K. Highly stable and efficient non-precious metal electrocatalysts of Mo-doped NiOOH nanosheets for oxygen evolution reaction. *Int. J. Hydrogen Energy* **2018**, *43*, 12140–12145, https://doi.org/10.1016/j.ijhydene.2018.04.181.
9. Hao, S.; Chen, L.; Yu, C.; Yang, B.; Li, Z.; Hou, Y.; Lei, L.; Zhang, X. NiCoMo Hydroxide Nanosheet Arrays Synthesized via Chloride Corrosion for Overall Water Splitting. *ACS Energy Lett.* **2019**, *4*, 952–959, https://doi.org/10.1021/acsenergylett.9b00333.
10. Guan, C.; Xiao, W.; Wu, H.; Liu, X.; Zang, W.; Zhang, H.; Ding, J.; Feng, Y.P.; Pennycook, S.J.; Wang, J. Hollow Mo-doped CoP nanoarrays for efficient overall water splitting. *Nano Energy* **2018**, *48*, 73–80, https://doi.org/10.1016/j.nanoen.2018.03.034.
11. Wang, D.; Li, Q.; Han, C.; Xing, Z.; Yang, X. When NiO@Ni Meets WS_2 Nanosheet Array: A Highly Efficient and Ultrastable Electrocatalyst for Overall Water Splitting. *ACS Central Sci.* **2017**, *4*, 112–119, https://doi.org/10.1021/acscentsci.7b00502.
12. Luo, X.; Shao, Q.; Pi, Y.; Huang, X. Trimetallic Molybdate Nanobelts as Active and Stable Electrocatalysts for the Oxygen Evolution Reaction. *ACS Catal.* **2018**, *9*, 1013–1018, https://doi.org/10.1021/acscatal.8b04521.
13. Xu, H.; Shang, H.; Wang, C.; Jin, L.; Chen, C.; Wang, C.; Du, Y. Three-dimensional open $CoMoO_x/CoMoS_x/CoS_x$ nanobox electrocatalysts for efficient oxygen evolution reaction. *Appl. Catal. B: Environ.* **2020**, *265*, 118605, https://doi.org/10.1016/j.apcatb.2020.118605.
